# Response of human periodontal ligament stem cells to IFN-γ and TLR-agonists

**DOI:** 10.1038/s41598-017-12480-7

**Published:** 2017-10-09

**Authors:** Oleh Andrukhov, Johanna Sang-A Hong, Olena Andrukhova, Alice Blufstein, Andreas Moritz, Xiaohui Rausch-Fan

**Affiliations:** 1 Division of Conservative Dentistry and Periodontology, University Clinic of Dentistry, Medical University of Vienna, Vienna, Austria; 2 Department of Biomedical Science, University of Veterinary Medicine, Vienna, Austria

## Abstract

Periodontal ligament stem cells similarly to the mesenchymal stem cells of other tissues possess immunomodulatory properties, which are regulated by different cytokines, particularly by interferon-γ (IFN-γ). In contrast, less information is provided about the effect of toll-like receptors ligand on immunomodulatory properties of these cells. In the present study we investigated the response of human periodontal ligament stem cells (hPDLSCs) in response to simultaneous stimulation with IFN-γ and toll-like receptor (TLR) agonists. The resulting expression of indoleamine-2,3-dioxygenase-1 (IDO-1), interleukin (IL)-6, IL-8 and monocyte chemotactic protein 1 (MCP-1) was investigated. The expression of IDO-1 was upregulated by IFN-γ in both gene and protein levels. TLR2 agonists Pam3CSK4 induced gene expression of IDO-1, but had no effect on protein expression. IFN-γ induced IDO-1 protein expression was further enhanced by Pam3CSK4. TLR-4 agonist *E. coli* LPS has no significant effect on neither basal nor IFN-γ induced IDO-1 protein expression. The production of IL-6, IL-8, and MCP-1 was induced by TLR agonists. Neither basal nor TLR agonists induced production of these proteins was affected by IFN-γ. Our data shows potential interaction between IFN-γ and TLR2 responses in hPDLSCs, which might be involved in regulation of immune response in inflammatory diseases, and particularly periodontitis.

## Introduction

Periodontitis is an inflammatory disease leading to the destruction of periodontal tissue and in worst cases to the tooth loss^1,2^. Periodontal disease is initiated by the shift of oral microbiota from healthy symbiotic to dysbiotic and is driven by immune response to pathogenic microorganisms^3,4^. Shift of oral microbiota results in activation of both innate and acquired immune systems, which is initially directed to eliminate overgrowing periodontal pathogens but also cause collateral host tissue damages. Inappropriate and dysregulated immune response is the main reason of tissue destruction and bone loss in periodontitis. The cellular mechanisms underlying the immune response in periodontitis are very complex and involve the interaction between bacteria, viruses and different types of host cells. The immune response is orchestrated by numerous cytokines, chemokines, and growth factors, which are produced by resident host cells^4^.

Mesenchymal stem cells (MSC) are recognized to modulate the immune system. The immunomodulatory ability is not characteristic for resting MSC and is activated by various inflammatory cytokines^5^. Studies of recent years revealed that IFN-γ plays a key role in the activation of immunomodulatory activity of MSC^6^. Interferon-γ is a cytokine, which is largely involved in the immune response in periodontal disease^7^. It is produced mainly by natural killer cells, activated CD4+ Th1 cells, and cytotoxic CD8+ cytotoxic T cell^8^. The activation of immunomodulatory activity of MSC is mainly associated with the upregulation of indoleamine-2,3-dioxygenase 1 (IDO-1) expression in MSC. IDO-1 is an enzyme which catalyses oxidative degradation of L-tryptophane, which results in the inhibition of local immune response^9,10^. The expression of IDO-1 is low in resting MSC and is strongly activated by IFN-γ^6^. In contrast to IFN-γ, much less is known about the effect of toll-like receptor (TLR) ligands on the expression of IDO-1 in MSC^11^.

It is recognized that dental tissues contains resident stem cells, which are similar to MSC isolated from bone marrow^12,13^. Human periodontal ligament stem cells (hPDLSCs) are resident MSC-like cells of periodontal tissue and might be involved in the immunomodulation and regulation of periodontal disease progression^14,15^. The exaxt mechanisms, by which hPDLSCs are involved in the progression of periodontal disease are not known, but recent study shows that hPDLSCs isolated from inflamed periodontal tissue exhibit impaired immunomodulatory properties^16^. Similarly to MSC from other sources, periodontal ligament stem cells express IDO-1 upon stimulation with IFN-γ^17^. A recent study shows that IL-12 upregulates the IDO-1 expression in human periodontal ligament cells through stimulation of IFN-γ production^18^. One study shows that LPS induces the expression of IDO-1 in periodontal ligament cells on both gene and protein levels^19^. However, the role of different bacterial components and TLR ligands in the activation of immunomodulatory ability of human periodontal ligament stem cells (hPDLSC) still remains to be clarified. It should be noted, that the role of hPDLSC in periodontitis cannot be attributed only to suppression of immune response. hPDLSC produces different inflammatory mediators in response to stimulation with bacterial LPS and synthetic TLR2 agonists^20,21^, which might stimulate immune response and leukocytes infiltration into periodontal tissue. Thus, the contribution of resident MSC to the immune response in periodontitis is rather complex and must be clarified by further studies.

Under conditions *in vivo*, cells are simultaneously exposed to different pro-inflammatory cytokines and bacterial components. The interaction between different signalling pathways is multifaceted and contributes into immune response^22,23^. It could be hypothesized that simultaneous stimulation of hPDLSC with IFN-γ and TLR agonists might reciprocally modify the cell response to each of these factors. However, this question is investigated rather poorly to date. TLR2 and TLR4 agonists are of especial interest in our study, because these receptors play a key role in the progression of periodontal disease^24,25^ and are activated by bacterial components such as lipopolysaccharide, peptidoglycan, lipoteichoic acid, bacterial lipoproteins. Therefore, in the present study we investigated the simultaneous effect of IFN-γ and TLR2 and TLR4 agonists on the expression of immunomodulatory protein IDO-1 and inflammatory mediators interleukin (IL) 6, IL-8, and MCP-1 in hPDLCS.

## Results

### The effect of IFN-γ, Pam3CSK4, and *E. coli* LPS on gene expression of IDO-1 in hPDLSCs

**Figure 1:**
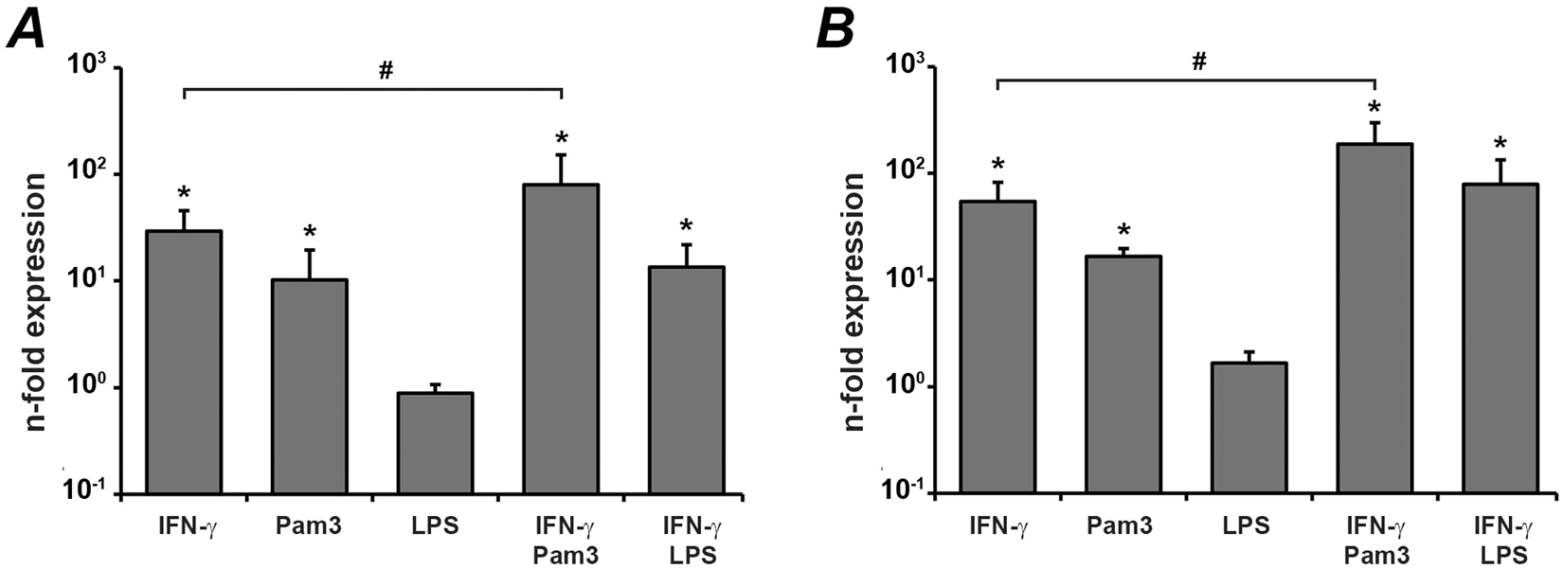
Effect of IFN-γ, Pam3CSK4, and *E. coli* LPS on the gene expression level of IDO-1 in hPdLSC. Cells were stimulated with IFN-γ (10 ng/ml), TLR2 agonist Pam3CSK4 (1 µg/ml), TLR4 agonist *E. coli* LPS (1 µg/ml), or combinations of IFN-γ and TLR agonists for either 6 h (**A**) or 48 h (**B**) and resulting gene expression of IDO-1 was measured by qPCR. Y-axis represents n-fold expression levels compared to unstimulated cells. Data are presented as mean ± s.e.m. of 6 different donors. *significantly different compared to control, p < 0.05. ^#^significantly different between groups, p < 0.05.

The effect of IFN-γ, Pam3CSK4, and *E. coli* LPS and their combinations on the gene expression levels of IDO-1 in human periodontal ligament stem? cells is shown in the Fig. 1. After 6 h stimulation, IFN-γ and Pam3CSK4 induced a significant increase in IDO-1 mRNA expression levels, whereas *E. coli* LPS hasd no significant effect on IDO-1 gene expression. Combination of IFNg-γ and PAMam3CSK4 induced significantly higher IDO-1 gene expression levels compared to stimulation with single stimuli. *E. coli* LPS had no significant effect on IFN-γ induced response. After 48 h stimulation, a significant increase in the IDO-1 gene expression levels was observed after stimulation with IFN-γ and Pam3CSK4. However, the response to IFN-γ was significantly higher than that to Pam3CSK4. Pam3CSK4 also enhanced the IFN-γ induced IDO-1 expression. *E. coli* LPS had effect on neither basal nor IFN-γ induced IDO-1 mRNA expression.

### The effect of IFN-γ, Pam3CSK4, and *E. coli* LPS on IDO-1 protein expression in hPDLSCs

**Figure 2:**
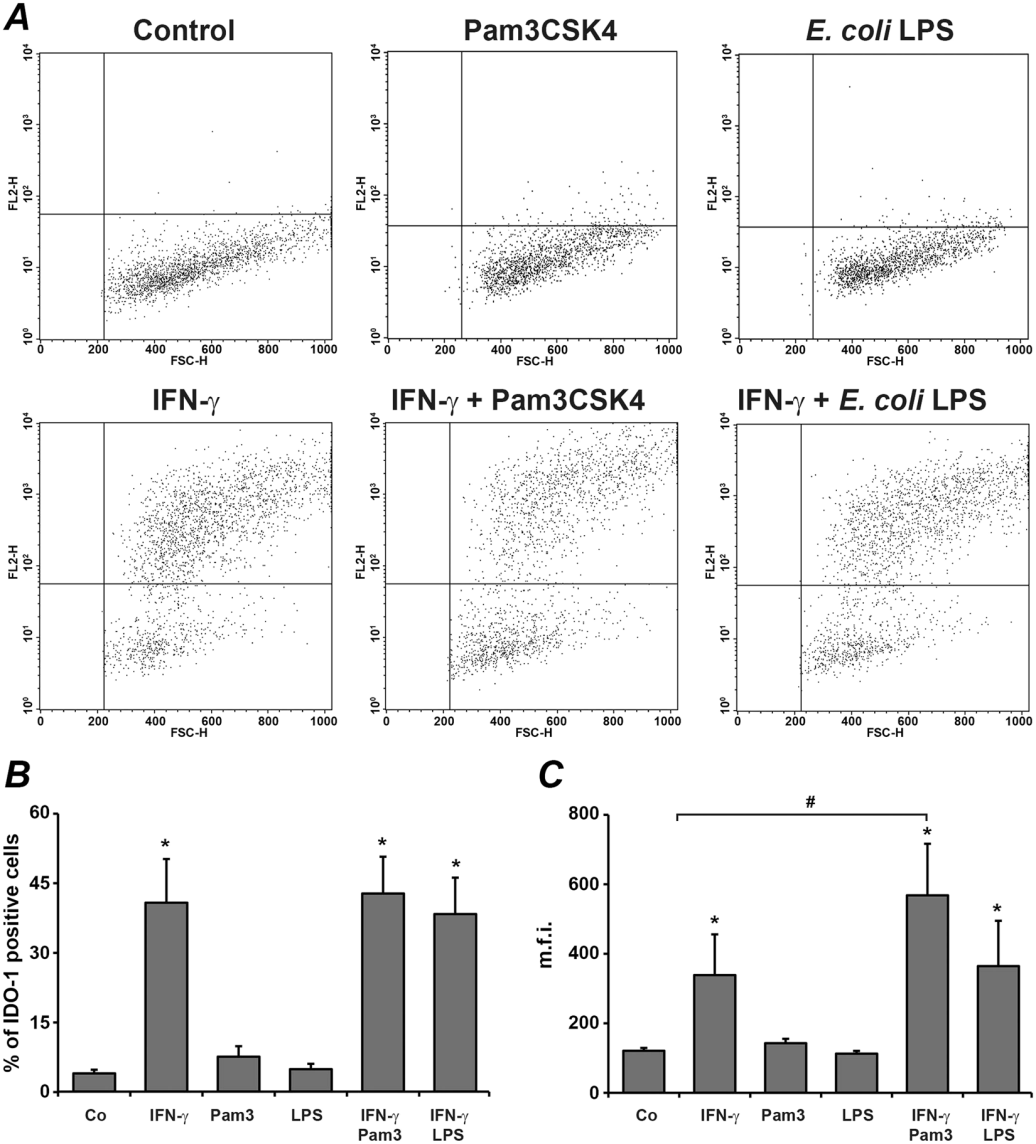
Effect of IFN-γ, Pam3CSK4, and *E. coli* LPS on intracellular expression of IDO-1 in human periodontal ligament stem cells. Cells were stimulated with IFN-γ (10 ng/ml), TLR2 agonist Pam3CSK4 (1 µg/ml), TLR4 agonist *E. coli* LPS (1 µg/ml), or combinations of IFN-γ and TLR agonists for 48 h. After stimulation, cells were stained intracellulary with anti IDO-1 antibody and proceeded to flow cytometry analysis. (**A**) original FACS dot plots of hPDLSCs stained intracellularly with anti-human IDO-1 antibody after stimulation with different stimuli. (**B**) percentage of hPDLSCs stained positively with anti-human IDO-1 antibody. (**C**) mean fluorescence intensity of hPDLSCs positively stained with anti-human IDO-1 antibody. Data are presented as mean ± s.e.m. of 6 different donors. *significantly different compared to control, p < 0.05. ^#^significantly different between groups, p < 0.05.

No significant changes of IDO-1 protein expression measured by intracellular staining with anti-IDO-1 antibody were detected in hPDLSC after stimulation with any stimuli for 6 h (data not shown). The results of flow cytometry analysis of hPDLSC stained intracellular with anti-IDO-1 antibody after 48 h stimulation with different stimuli are shown in the Fig. 2. Original records showed the appearance of numerous IDO-1-positively stained cells after stimulation with IFN-γ, but not after stimulation with Pam3CSK4 or *E. coli* LPS (Fig. 2A). The proportion of positively stained cells was significantly higher after IFN-γ stimulation compared to control. Neither Pam3CSK4 nor *E. coli* LPS had significant effect on the proportion of positively stained cells induced by IFN-γ. However, the mean fluorescence intensities of IDO-1 positive cells were significantly higher after stimulation with IFN-γ and Pam3CSK4 compared to stimulation with IFN-γ only.

### The effect of IFN-γ, Pam3CSK4, and *E. coli* LPS on the gene expression levels of IL-6, IL-8 and MCP-1 in hPDLSCs

**Figure 3:**
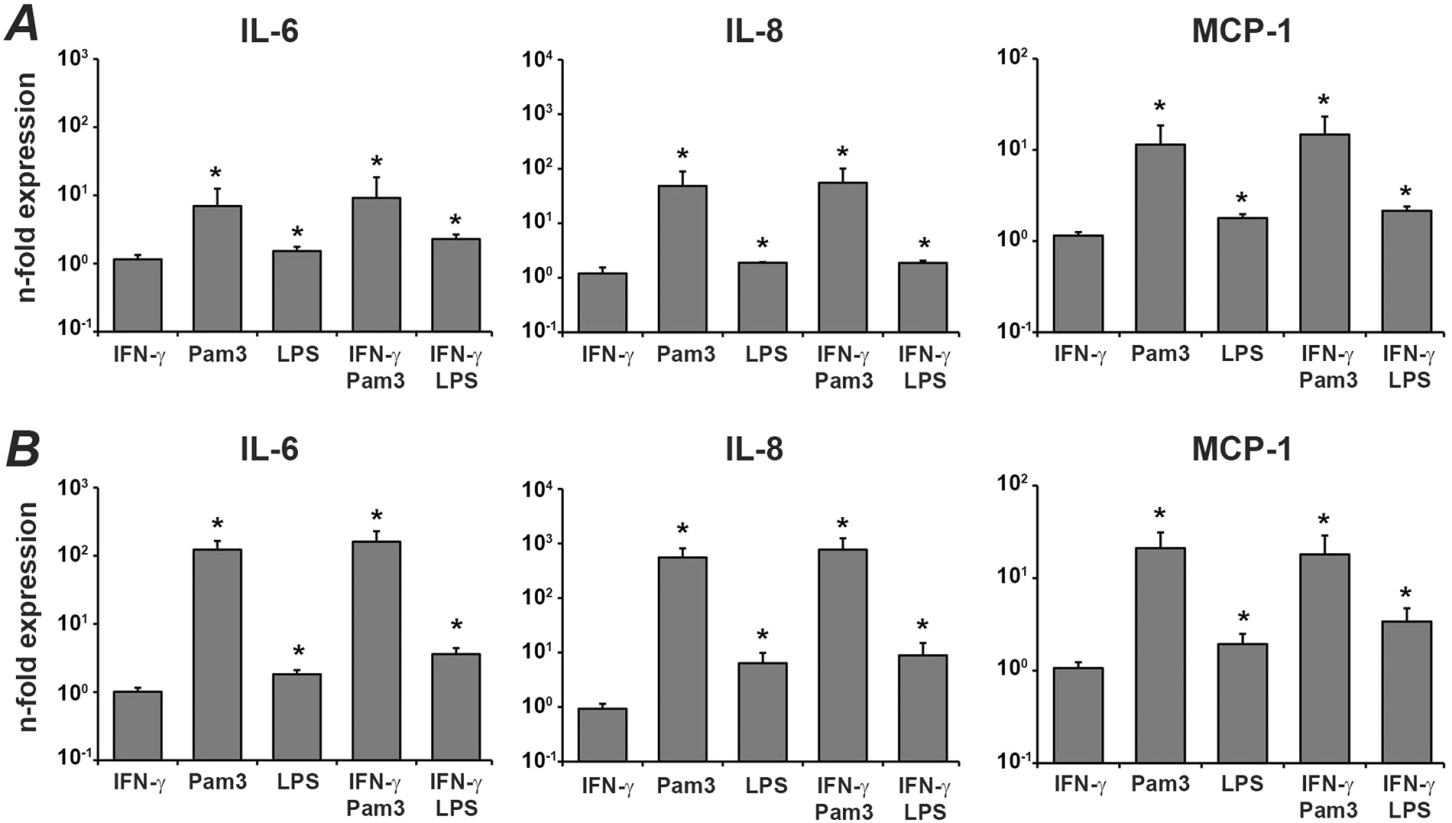
Effect of IFN-γ, Pam3CSK4, and *E. coli* LPS on the gene expression level of IL-6, IL-8, and MCP-1 in hPDLSC. Cells were stimulated with IFN-γ (10 ng/ml), TLR2 agonist Pam3CSK4 (1 µg/ml), TLR4 agonist *E. coli* LPS (1 µg/ml), or combinations of IFN-γ and TLR agonists for either 6 h (**A**) or 48 h (**B**) and resulting expression of IL-6, IL-8, and MCP-1 was measured by qPCR. Y-axis represents the n-fold expression levels of target gene in relation to unstimulated cells (n = 1). Data are presented as mean ± s.e.m. of 6 different donors. *significantly different compared to control.

The effect of different stimuli on the gene expression levels of IL-6, IL-8, and MCP-1 is shown in the Fig. 3. After 6 h, the expression of all three proteins was significantly increased by Pam3CSK4 and *E. coli* LPS. The response of hPDLSCs to Pam3CSK4 was significantly higher than that to *E. coli* LPS. IFN-γ had no significant effect on the gene expression of all these proteins. This was true for basal expression as well as for TLR agonist induced expression. Qualitatively similar results were observed after 48 h stimulation.

### The effect of IFN-γ, Pam3CSK4, and *E. coli* LPS on the production of IL-6, IL-8 and MCP-1 by hPDLSCs

**Figure 4:**
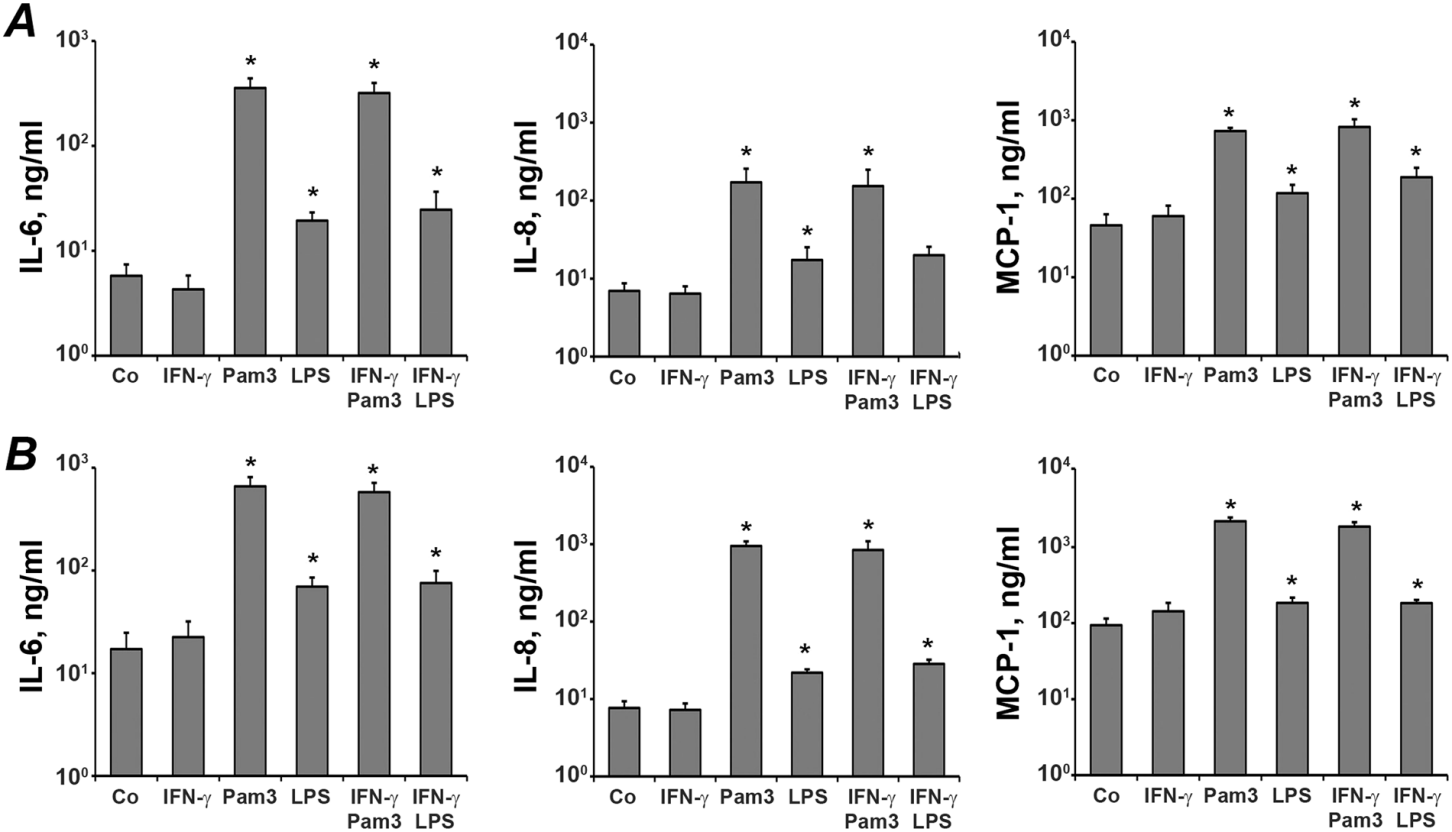
Effect of IFN-γ, Pam3CSK4, and *E. coli* LPS on production of IL-6, IL-8, and MCP-1 protein by hPDLSC. Cells were stimulated with IFN-γ (10 ng/ml), TLR2 agonist Pam3CSK4 (1 µg/ml), TLR4 agonist *E. coli* LPS (1 µg/ml), or combinations of IFN-γ and TLR agonists for either 6 h (**A**) or 48 h (**B**) and concentration of IL-6, IL-8, and MCP-1 protein in conditioned media were measured by ELISA. Data are presented as mean ± s.e.m. of 6 different donors. *significantly different compared to control.

The production of IL-6, IL-8, and MCP-1 in response to stimulation with different stimuli is shown in the Fig. 4. The results on protein production were generally in agreement with data obtained by gene expression analysis. Production of IL-6, IL-8, and MCP1 was enhanced by Pam3CSK4 and *E. coli* LPS, but not by IFN-γ. The effect of Pam3CSK4 on protein production was significantly higher than that of *E. coli* LPS. IFN-γ had also no effect on the protein production induced by TLR agonists.

## Discussion

In the present study we investigated the effect of IFN-γ and/or TLR agonists on the expression of IDO-1, IL-6, IL-8, and MCP-1 in primary human periodontal ligament stem cells. These proteins might be involved in immunomodulatory function of hPDLSCs. Particularly, IDO-1 is the major protein associated with immune suppression, whereas IL-6, IL-8, and MCP-1 are pro-inflammatory proteins^26^. Cells were stimulated with different stimuli for either 6 h or 48 h. These time points were selected because of different time course of cell response and temporal gene expression. The response to TLR agonist and production of pro-inflammatory proteins occurs within first hours after stimulation, whereas an increased expression of IDO-1 upon stimulation with IFN-γ is observed after prolonged stimulation up to 72 h^27–29^.

Our data clearly show that activation of IDO-1 expression in hPDLSCs is induced by IFN-γ on both gene and protein level. Interestingly, IDO-1 gene expression was significantly enhanced by IFN- after both 6 h and 48 h stimulation, whereas protein expression was increased only after 48 h stimulation. A mismatch between gene and protein expression could be explained due to several mechanisms regulating mRNA stability and translation process, which might be activated by IFN-γ^30^. The fact, that IDO-1 protein expression is increased only after prolonged stimulation is in line with data obtained on MSC isolated from other sources. In a study on human bone marrow MSC, IFN-γ induced IDO-1 expression only after stimulation for more than 12 h, whereas the response after 3-6 h stimulation was rather small^31^. Time used for IDO-1 induction in MSC usually varies from 18 h to 72 h^28–30,32^. A fact that IDO-1 expression is increased only after prolonged stimulation could be important for timely appropriate inhibition of inflammatory response, which should not happen too early in order to not interfere with pathogen elimination.

TLR2 and TLR4 agonists augmented only gene expression of IDO-1 but are not able to affect its protein levels. Inability of TLR2 and TLR4 agonist to induce IDO-1 protein expression is in line with a common opinion that activation of these receptors induces pro-inflammatory MSC phenotype and is not able to induce IDO-1 protein expression^11^. Our data are in line with a a recent study on dental pulp cells showing that only IFN-γ but not Pam3CSK4 and *E. coli* LPS might induce IDO-1 protein expression^33^. Surprisingly, our data about the effect of LPS on IDO-1 expression are in contrast with a previous study on periodontal ligament cells^19^, in which *E. coli* LPS was shown to increase IDO-1 mRNA expression, IDO-1 protein expression measured by western blot, and IDO-1 enzymatic activity. The reason for the discrepancies between these data and the results of our study are not entirely clear. Differences in cells isolation procedure, cell culture, stimulation protocol, and cell source could be considered as potential explanation. In our study hPDLSCs were isolated from healthy young donors aging from 16–19 years, experiments were performed in FCS-free DMEM. In a study of Moon *et al*. experiments were performed in RPMI-1640 medium and the information about donor age is missing^19^.

Although TLR2 agonist Pam3CSK4 does not induce IDO-1 protein expression, it might enhance IDO-1 expression induced by IFN-γ. Interestingly, Pam3CSK4 did not influence the proportion of IDO-1 positive cells induced by IFN-γ, but increased fluorescence intensity of IDO-1 positive cells. This can be interpreted as TLR2 agonist increases IDO-1 protein levels induced by IFN-γ. In contrast to Pam3CSK4, TLR4 agonist *E. coli* LPS had no significant effect on the IFN-γ induced IDO-1 expression. These data are only partially in agreement with previous study on dental pulp cell showing that both Pam3CSK4 and *E. coli* LPS increase IFN-γ induced IDO-1 protein expression^33^. The discrepancy concerning the effect of TLR4 agonist *E. coli* could be due to different properties of mesenchymal stem cells isolated from different sources. As shown by one study, TLR4 agonist LPS differently regulate immunomodulatory properties of MSC isolated from dental pulp and dental follicle^34^. Our recent study shows that TLR4 agonist *E. coli* LPS induces rather weak response in hPDLCs compared to Pam3CSK4^21^. In the present study, the effect of Pam3CSK4 on the production of IL6, IL-8, and MCP-1 by hPDLSCs was also significantly higher than that of *E. coli* LPS.

An important observation of our study is that an increase in the IDO-1 expression on mRNA level is not always accompanied by an increase in the protein expression. Particularly, IDO-1 mRNA expression was increased after 6 h stimulation with IFN-γ, but no changes in the protein expression was observed. Furthermore, TLR2 agonist Pam3CSK4 induced a significant increase in the IDO-1 mRNA expression, but has no effect on protein expression. The uncoupling between gene and protein expression levels of IDO-1 is also suggested by recent study of the effect of intereukin 12 on the immunomodulatory properties of periodontal ligament stem cells^18^. Here, IL-12 induces IFN-γ mediated increase in IDO-1 mRNA expression already after 24 h stimulation, whereas an increase in the IDO-1 activity is observed only after 7 days stimulation. Thus, measurements of IDO-1 protein expression and/or its activity are necessary to conclude about potential immunomodulatory properties of mesenchymal stem cells.

The production of IL-6, IL-8, and MCP-1 by periodontal ligament cells was significantly enhanced by TLR2 and TLR4 agonists but was not influenced by IFN-γ. Moreover, IFN-γ did not influence the production of IL-6, IL-8, and MCP-1 induced by TLR agonists. Thus, it seems that there is no synergistic effect between TLR agonists and IFN-γ in hPDLSCs. This question is controversy discussed by previous studies. In agreement with our data, IFN-γ has no effect on either basal or LPS-induced IL-6 and IL-8 production in both primary bone marrow MSC and immortalized MSC cell line V54/2^35^. No effect of IFN-γ on IL-6 production is observed by study on human periodontal ligament cells^36^. In contrast, in a study on human periodontal ligament cell IFN-γ at concentration 100 ng/ml induces a significant increase in the IL-6 and IL-8 production upon 24 h stimulation^37^. In a study on dental pulp cells shows that IFN-γ enhances the IL-6 production by these cells in response to stimulation with Pam3CSK4 and *E. coli* LPS^33^. Another study on bone marrow MSC shows that IFN-γ enhances IL-6 and IL-8 production in response to LPS^27^. The discrepancies between different studies could be due to several factors such as donors’ characteristics, cell source, IFN-γ concentration and stimulation protocol.

We found that TLR2 and TLR4 agonists differently affect IFN-γ induced response in hPDLSCs. Moreover, the substantial quantitative differences in IL-6, IL-8 and MCP-1 production were observed between Pam3CSK4 and *E. coli* LPS. A reason for such difference in the hPDLSCs response to TLR2 and TLR4 agonists is not currently known and must be investigated in further studies. There are some differences in the intracellular signalling between TLR2 and TLR4 signalling pathways^38^. Upon activation, both TLR2 and TLR4 recruit intracellular adaptor protein MyD88, which plays a key role in the intracellular signalling^39^. Recruitment of MyD88 to TLRs requires MyD88 adaptor-like (Mal) protein^40^. TLR4 also activates MyD88-independent signalling pathway. Studies of recent years shows that Mal might trigger also MyD88 independent signalling upon TLR2 activation^41^. In addition, at high TLR2 agonists at high concentrations might induce Mal-independent response^42,43^. The intracellular pathways involved in TLR2 and TLR4 signalling in human MSC, and particularly in hPDLSCs, should be further identified.

## Methods

### Cell Culture and Reagents

Primary periodontal ligament cells were isolated from periodontally healthy patients undergoing routine extraction of their third molar teeth. All donors were Caucasians, 16–19 years old, generally healthy, non-smokers. Four donors were females, two donors were males. Patients (or their parents) were informed in detail before the surgical procedures and gave their written agreement. The study protocol was approved by the Ethics Committee of the Medical University of Vienna. All experiments were performed in accordance with “Good Scientific Practice” guidelines of Medical University of Vienna and Declaration of Helsinki. Periodontal ligament tissue was scraped from the teeth root surface with a scalpel, cut into small pieces and digested by collagenase/dispase (Sigma, St. Louis, MO, USA) for 30 min at 37 °C. Cells were cultured in Dulbecco’s modified Eagle’s medium (DMEM), supplemented with 10% fetal bovine serum (FBS), streptomycin (S) (50 µg/ml) and penicillin (P) (100 U/ml) under humidified air atmosphere of 5% CO_2_ at 37 °C. Cells isolated from 6 different donors from passage levels 3–6 were used in the experiments. The expression of specific surface markers as well as the differentiation ability was proved as described in previous study^21^. Commercially available interferon-γ, *E. coli* LPS (ultrapure preparation), TLR-2 agonist Pam3CSK4 (both Invivogen, San-Diego, USA), human soluble CD14 was purchased from Peprotech (St. Louis, MO, USA).

### Stimulation protocol

Cells were seeded in a 6-well plate at a density of 2.5 × 10^5^ cells per well. After 24 h, medium was changed to serum-free DMEM with 1% P/S. Cells were stimulated with one of the following stimuli: human recombinant IFN-γ (10 ng/ml); TLR2/1 agonist Pam3CSK4 (1 µg/ml); *E. coli* LPS (1 µg/ml); IFN-γ (10 ng/ml) and Pam3CSK4 (1 µg/ml); IFN-γ (10 ng/ml) and *E. coli* LPS (1 µg/ml). *E. coli* LPS was applied in combinations with soluble CD14 (250 ng/ml), since our recent study shows that CD14 is crucial for hPDLSC response to bacterial LPS^21^. After stimulation, the cellular mRNA expression levels of IDO-1, IL-6, IL-8, and MCP-1 was measured by qPCR. The protein expression level of IDO-1 in hPDLSCs was assessed by flow cytometry. The levels of IL-6, IL-8, and MCP-1 protein in conditioned media were determined by commercially available ELISA Ready-Set-Go! kits (eBioscience, San Diego, CA, USA).

### Quantitative PCR

The mRNA expression levels of IL-6, IL-8, MCP-1, and IDO-1 were determined by qPCR as described previously^44,45^, taking the β-actin encoding gene as internal reference. Isolation of mRNA and transcription into cDNA was performed using the TaqMan Gene Expression Cells-to-CT kit (Ambion/Applied Biosystems, Foster City, CA, USA), which provides good accuracy and superior sensitivity of gene-expression analysis^46^. qPCR was performed on an ABI StepOnePlus device (Applied Biosystems) in paired reactions using the Taqman gene expression assays with following ID numbers (all from Applied Biosystems): IL-6, Hs00985639_m1; IL-8, Hs00174103_m1; MCP-1, Hs00234140_m1; IDO-1, Hs00984148_m1; GAPDH, Hs99999905_m1. qPCR reactions were performed in triplicate in 96-well plates using the following thermocycling conditions: 95 °C for 10 min; 40 cycles, each for 15 s at 95 °C and at 60 °C for 1 min. The point at which the PCR product was first detected above a fixed threshold (cycle threshold, C_t_), was determined for each sample. Changes in the expression of target genes were calculated using the 2^−ΔΔCt^ method, where ΔΔC_t_ = (C_t_^target^ − C_t_^GAPDH^)_sample_ − (C_t_^target^ − C_t_^GAPDH^)_control_, taking an untreated sample as a control.

### Measurements of IDO-1 protein expression by flow cytometry

Cells were detached with accutase (eBioscience, San Diego, CA, USA) and transferred into FACS buffer (3% FCS, 0.9% NaN_3_ in PBS). 5 × 10^5^ cells were fixed by incubation in FACS buffer containing 4% formaldehyde for 15 min and permeabilized by incubation in FACS buffer containing 1% Triton X-100 for 20 min. Afterwards, cells were resuspended in 50 µl of FACS buffer, mixed thoroughly with 5 µl of phycoerythrin-conjugated mouse anti-human IDO-1 antibody (clone eyedio, eBioscience, San Diego, CA, USA). Cells treated with similar procedure and stained with phycoerythrin-conjugated mouse IgG1 K immunoglobulin isotype control were used as reference. The incubation was performed in dark place for 20 min. After incubation, the cells were washed twice with ice-cold FACS buffer, re-suspended in 300 µl of FACS buffer and analysed for IDO-1 expression. Analysis was performed with a flow cytometer (FACScan, Becton Dickinson, San Jose, CA, USA) equipped with an argon laser tuned at 488 nm. Cell counting was limited by 5000 events.

### Statistical analysis

The normal distribution of all data was tested with Kolmogorov-Smirnov test. After confirming normal distribution, the statistical differences between different groups were analysed by one-way analysis of variance (ANOVA) for repeated measures followed by t-test. All statistical analyses were performed using statistical program SPSS 21.0 (SPSS, Chicago, IL, USA). Data are expressed as mean ± S.E.M. Differences were considered to be statistically significant at p < 0.05.
